# PD161 Distribution Patterns And Economic Assessments Of Gaucher Disease Therapies In Brazil: A National Health System Analysis (1999 to 2022)

**DOI:** 10.1017/S0266462324003908

**Published:** 2025-01-07

**Authors:** Marcus Carvalho Borin, Francisco de Assis Acurcio, Juliana Alvares-Teodoro, Augusto Guerra

## Abstract

**Introduction:**

Gaucher disease, an inherited lysosomal storage disorder, requires chronic management with enzyme replacement therapies (ERTs). In Brazil, the Unified Health System (SUS) plays a pivotal role in providing access to such treatments. This study aimed to analyze the distribution and associated costs of medications for Gaucher disease within the SUS, offering a comprehensive view of resource allocation over 23 years.

**Methods:**

Utilizing the TabNet system from the Brazilian Health Ministry, medication dispensation data from 1999 to 2022 were analyzed. In addition, annual and total expenditures on imiglucerase, miglustat, and taliglucerase alfa were evaluated using the Ambulatory Information System and the Hospital Information System databases for a cohort of patients from 2000 to 2015. Demographic factors such as sex, age, self-declared skin color, body mass index, and area of residence were correlated with spending patterns. Trends were contextualized with events that could potentially affect medication availability, such as ministry alerts and regulatory changes.

**Results:**

The dispensation analysis revealed a fluctuating pattern in medication distribution over the study period. The data revealed a peak in imiglucerase dispensation in the mid-2000s, followed by a stark decrease after 2010 that coincided with global shortages. Total costs from 2000 to 2015 reached USD1.138 billion, with annual expenditures averaging USD120,631.15. After 2010 there was a diversification in therapy utilization, with an increase in alternative treatments such as miglustat and taliglucerase alfa.

**Conclusions:**

The study reveals a significant financial burden on the SUS from Gaucher disease treatments and demographic disparities. Trends in the dispensation and costs of ERTs within the SUS are a direct response to drug availability and regulatory actions, with adoption of alternative ERTs after 2010 demonstrating the system’s flexibility. Strategic health policy planning is vital for treatment sustainability and affordability.

